# Characterization of a spontaneous mouse model of mild, accelerated aging via ECM degradation in emphysematous lungs

**DOI:** 10.1038/s41598-023-37638-4

**Published:** 2023-07-03

**Authors:** Ryosuke Tanino, Yukari Tsubata, Takamasa Hotta, Tamio Okimoto, Yoshihiro Amano, Mayumi Takechi, Tetsuya Tanaka, Tomomi Akita, Mamiko Nagase, Chikamasa Yamashita, Koichiro Wada, Takeshi Isobe

**Affiliations:** 1 Department of Internal Medicine, Division of Respiratory Medicine and Medical Oncology, Faculty of Medicine, Shimane University, 89-1 Enya, Izumo, Shimane 693-8501 Japan; 2 Department of Experimental Animals, Interdisciplinary Center for Science Research, Organization for Research and Academic Information, Shimane University, Izumo, Japan; 3 Department of Human Nutrition, Faculty of Contemporary Life Science, Chugoku Gakuen University, Okayama, Japan; 4 Department of Pharmaceutics and Drug Delivery, Faculty of Pharmaceutical Sciences, Tokyo University of Science, Noda, Japan; 5 Department of Organ Pathology, Faculty of Medicine, Shimane University, Izumo, Japan; 6 Department of Pharmacology, Faculty of Medicine, Shimane University, Izumo, Japan

**Keywords:** Chronic obstructive pulmonary disease, Experimental models of disease

## Abstract

Emphysema limits airflow and causes irreversible progression of chronic obstructive pulmonary disease (COPD). Strain differences must be considered when selecting mouse models of COPD, owing to disease complexity. We previously reported that a novel C57BL/6JJcl substrain, the Mayumi-Emphysema (ME) mouse, exhibits spontaneous emphysema; however, the other characteristics remain unknown. We aimed to characterize the lungs of ME mice and determine their experimental availability as a model. ME mice had a lower body weight than the control C57BL/6JJcl mice, with a median survival time of ~80 weeks. ME mice developed diffused emphysema with respiratory dysfunction from 8 to 26 weeks of age, but did not develop bronchial wall thickening. Proteomic analyses revealed five extracellular matrix-related clusters in downregulated lung proteins in ME mice. Moreover, EFEMP2/fibulin-4, an essential extracellular matrix protein, was the most downregulated protein in the lungs of ME mice. Murine and human EFEMP2 were detected in the pulmonary artery. Furthermore, patients with mild COPD showed decreased EFEMP2 levels in the pulmonary artery when compared to those without COPD. The ME mouse is a model of mild, accelerated aging with low-inflammatory emphysema and respiratory dysfunction that progresses with age and pulmonary EFEMP2 decrease, similar to that observed in patients with mild COPD.

## Introduction

Chronic obstructive pulmonary disease (COPD) is a severe respiratory disease characterized by chronic bronchitis and emphysema. It is the third leading cause of death worldwide^1^, due to the lack of curative treatment^2^. Emphysema is characterized by abnormally large air spaces caused by the destruction of the alveolar walls and microvasculature, which contributes to progressive and irreversible airflow obstruction^3^. COPD is also a complex heterogenous disease linked to aging^4^ that is frequently associated with cachexia, muscular atrophy, and vascular diseases^5–7^. Although the inhalation of tobacco smoke is a major risk factor for COPD^8–10^, only a subset of smokers develop the disease phenotypes of COPD^11,12^. Combinations of various intrinsic and extrinsic factors, such as environmental pollutants, lung microflora, and impaired lung growth^12,13^, are likely to heterogeneously develop airway remodeling.

Preclinical studies aimed at developing COPD therapies have employed various animal models of emphysema induced by lung-damaging agents or spontaneous development^14–17^. Ideal animal models of COPD/emphysema should have characteristics that reflect the phenotypes seen in humans, with the potential for experimental-term intervention. However, the animal models are limited in their ability to reproduce the pathophysiological and molecular biological characteristics of humans, and the differences among models are critical for selecting models that appropriately mimic the target phenotypes of COPD^18^. We previously reported that the Mayumi-Emphysema (ME) mouse^19^, a novel naturally occurring substrain originating from the C57BL/6JJcl (B6) strain, exhibits emphysematous lungs without any extrinsic stimulus. However, other disease characteristics of ME mice remain unclear. Understanding the disease characteristics of ME mice is a prerequisite for their use in disease models, and will enhance the utility of studies on lung disorders.

The pathological structure of the distal airways relies greatly on the extracellular matrix (ECM)^20,21^, which consists of elastic fibers and collagen molecularly assembled by ECM proteins. The ECM provides the elastic structural scaffold for the fragile architecture of cells in the lung alveoli and pulmonary vascular system^22,23^. Regardless of disease origin, emphysematous alveolar destruction and arterial deficiency involve improper ECM protein degradation and synthesis^24^. Hence, lung cells and ECM proteins in the more fragile components are presumably lost in the early stages of emphysema development and may play a pivotal role. Therefore, we hypothesized that emphysematous lungs in patients with COPD and ME mice would have common molecular characteristics.

This study was designed to comprehensively evaluate ME mice and identify the molecular characteristics of their lungs via phenotypic, pathological, and proteomic characterization in comparison with control B6 mice. We also compared lung specimens from patients with or without COPD. The results of this study should provide an approach for further research on lung diseases linked to aging.

## Results

### ME mice survive more than a year with defective lungs and low body weights

**Figure 1:**
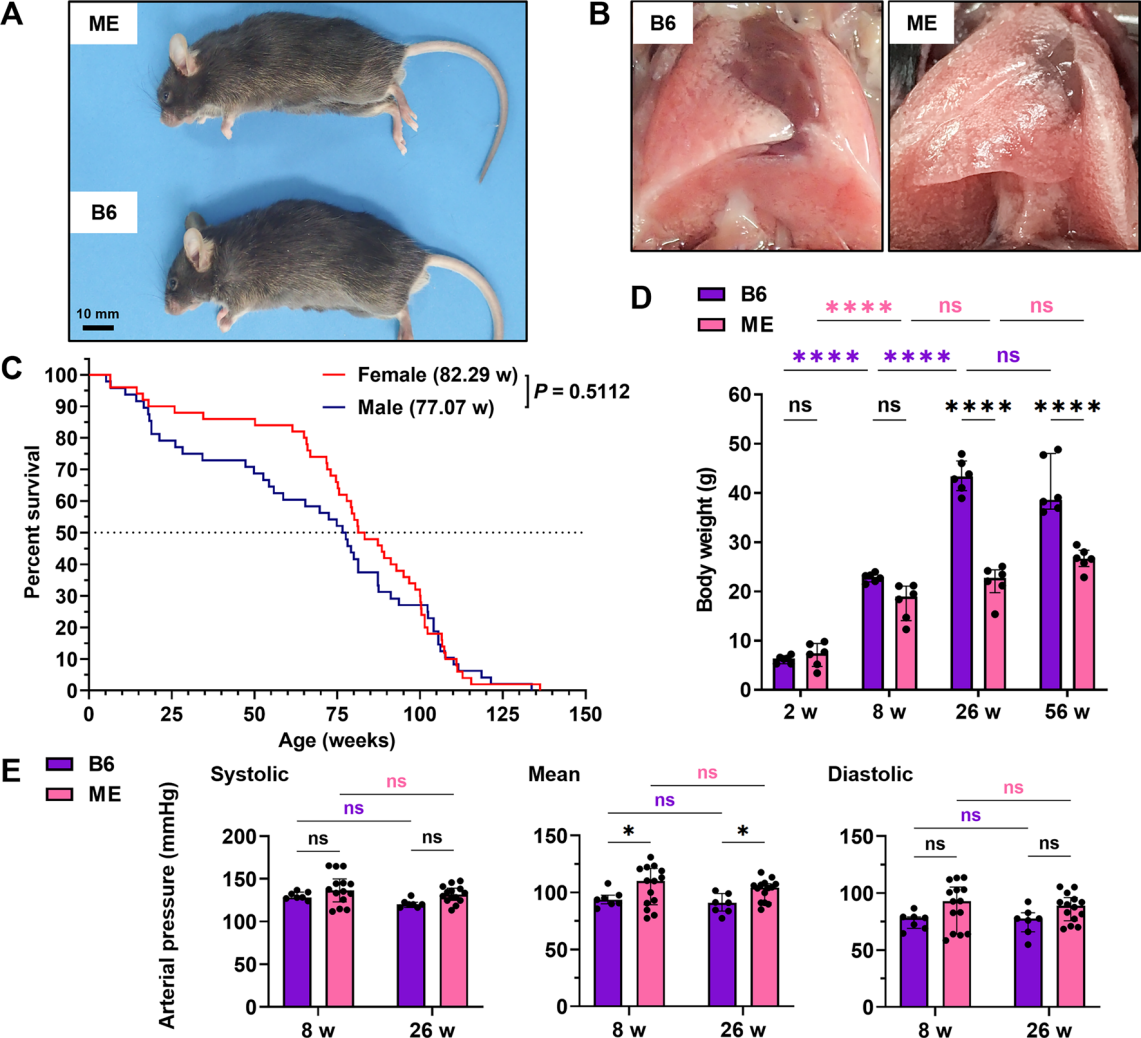
Phenotypic features of the Mayumi-Emphysema mouse (ME) model when compared to those of the parental strain, C57BL/6JJcl (B6). (**A**) Comparison of the appearance between B6 and ME mice. Scale bar, 10 mm. (**B**) The PBS-filled lungs of middle-aged ME mice were seemingly more translucent than those of B6 mice. (**C**) The survival curve of ME mice (female, n = 50; male, n = 48, log-rank test). (**D**) The bodyweight of ME mice was lower than that of B6 mice at 26 and 56 weeks of age. Data are presented as the median with interquartile range (n = 6 per group, two-way ANOVA and Sidak’s test for multiple comparisons). (**E**) Blood pressure of the tail vein. Data are presented as the median with interquartile range (B6, n = 7; ME, n = 14, two-way ANOVA and Sidak’s test for multiple comparisons). ns, not significant. *P < 0.05 and ****P < 0.0001.

For breeding and experimental use, ME mice are viable and fertile, and can be maintained under standard housing conditions. ME mice had slightly smaller eyes and bodies than B6 mice (Fig. 1A). The PBS-filled lungs of B6 and ME mice are shown in Fig. 1B. The lungs of aged (56-week-old) ME mice had more transparent regions after intrabronchial PBS injection than those of B6 mice. To evaluate the available experimental period, we assessed the survival time of ME mice. The mean survival time of ME mice was approximately 80 weeks (Fig. 1C), which was 40% lower than that of B6 mice, inferred from a large survival data study of mouse strains^25^. No significant difference was observed in survival time between the male and female ME mice (Fig. 1C). ME mice gained body weight in a manner that was similar to B6 mice until 8 weeks of age (Fig. 1D). However, after eight weeks, the body weight stopped increasing. Thus, 26- and 56-week-old mice had significantly lower body weights than the age-matched B6 mice (Fig. 1D). To evaluate systemic vascular function, we measured the blood pressure in B6 and ME mice. The mean blood pressure was slightly higher in the ME mice than in B6 mice (Fig. 1E). No characteristics of other diseases or specific causes of death were observed in the ME mice. ME mice showed systemic vulnerabilities in body weight and survival time, and lung abnormality was a phenotypic hallmark of ME mice.

### Aging impairs the respiratory function in ME mice

**Figure 2:**
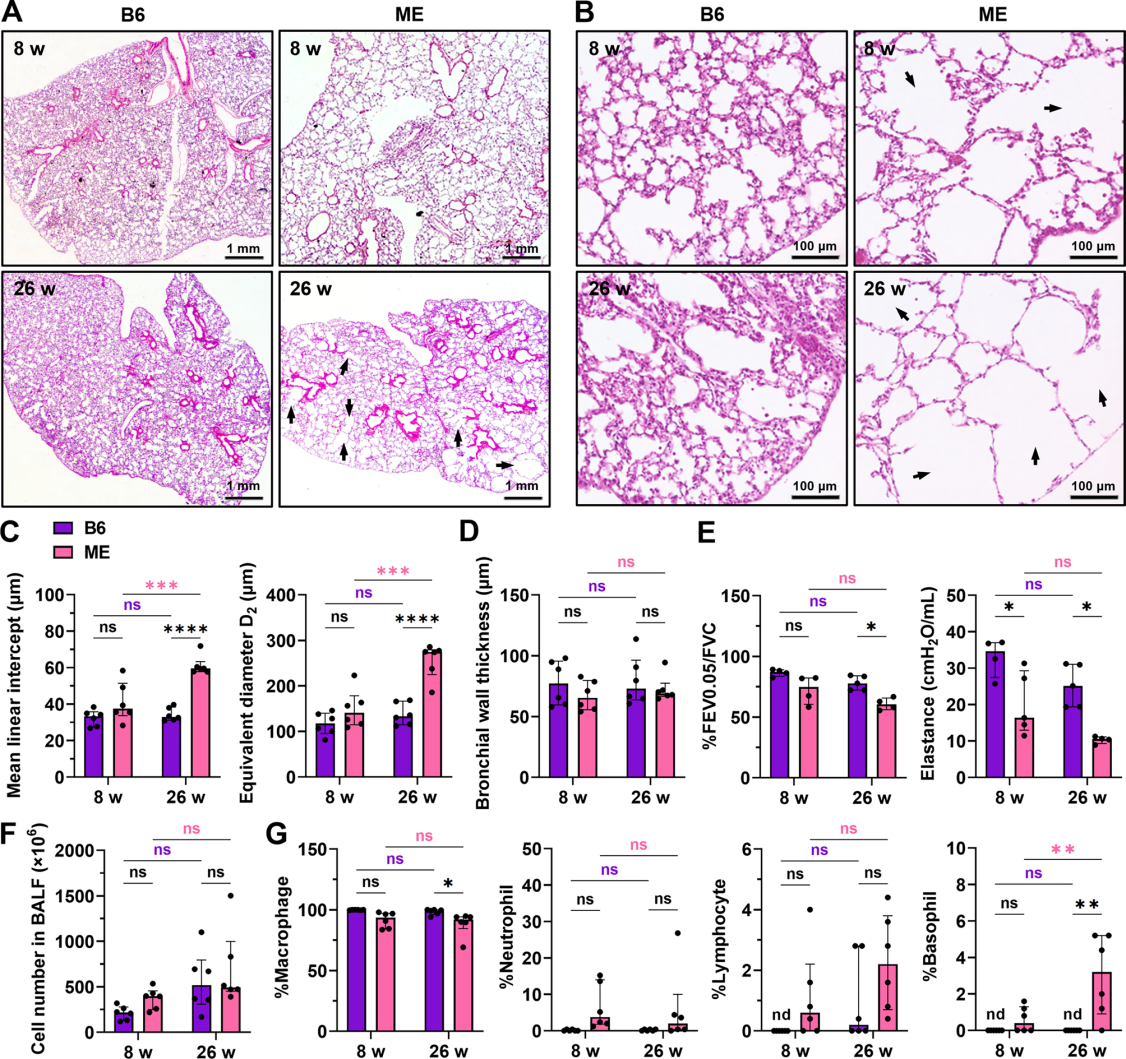
ME mice present with spontaneous emphysema progression and a decrease in respiratory function. (**A** and** B**) Histological changes in the lung at 8 and 26 weeks of age in B6 or ME mice. Arrows indicate prominent enlarged airspaces. Scale bars, (**A**) 1 mm and (**B**) 100 μm. (**C** and **D**) Histological quantification revealed airspace enlargement with aging in ME mice. (**C**) Mean linear intercept and equivalent airspace diameter D_2_ of lung sections from B6 and ME mice. Data are presented as the median with interquartile range (n = 6 per group, two-way ANOVA and Sidak’s test for multiple comparisons). (**D**) There was no difference in bronchial wall thickness between B6 and ME mice. Data are presented as the median with interquartile range (n = 6 per group, two-way ANOVA and Sidak’s test for multiple comparisons). (**E**) Respiratory function tests. Forced expiratory volume in 0.05 s per forced vital capacity (FEV0.05/FVC) decreased from 26 weeks. The tissue elastance was low in ME mice at 8 and 26 weeks of age. Data are presented as the median with interquartile range (n = 4–5 per group, two-way ANOVA and Tukey’s test for multiple comparisons). (**F** and **G**) Evaluation of intrapulmonary white blood cells in the bronchoalveolar lavage fluid (BALF). (**F**) Total number of white blood cells in BALF. No significant difference was observed in BALF cell numbers between ME and age-matched B6 mice. Data are presented as the median with interquartile range (n = 6 per group, two-way ANOVA and Sidak’s test for multiple comparisons). (**G**) The population of each white blood cell type in BALF. ME mouse BALF had fewer macrophages and more basophils than B6 mouse BALF at 26 weeks. Data are presented as the median with interquartile range (n = 6 per group, two-way ANOVA and Sidak’s test for multiple comparisons). ns, not significant; nd, not detected. *P < 0.05, **P < 0.01, ***P < 0.001, and ****P < 0.0001.

Next, we assessed histological lung disruptions in 8- and 26-week-old ME mice, which are convenient for experimental use as young and mature adults (Fig. 2A,B). Lung sections of 8-week-old mice showed partial emphysema, whereas those of 26-week-old mice showed diffused emphysema similar to panlobular emphysema, when compared to the lung sections of B6 mice. To examine whether these differences were significant and age-dependent, we measured the mean linear intercept (MLI) and equivalent airspace diameter D_2_. The MLI and D_2_ did not increase significantly in 8-week-old ME mice, but increased in 26-week-old ME mice (Fig. 2C). Although emphysema progressed in 26-week-old ME mice, bronchial wall thickening was not observed (Fig. 2D). Consistent with the histological differences, forced expiratory volume in 0.05 s per forced vital capacity (FEV0.05/FVC) of 8-week-old ME mice was not different from that of B6 mice, whereas 26-week-old ME mice had a lower FEV0.05/FVC than the 26-week-old B6 mice (Fig. 2E). In addition, tissue elastance in ME mice was significantly lower than that in the age-matched B6 mice (Fig. 2E). Bronchoalveolar lavage fluid (BALF) was collected from 8- and 26-week-old mice. ME mice exhibited no difference in BALF cell numbers from that observed in B6 mice (Fig. 2F). ME mice exhibited decreased macrophage counts in BALF when compared to the B6 mice at 26 weeks (Fig. 2G). Moreover, the number of basophils increased in the BALF of ME mice at 26 weeks (Fig. 2G). However, no differences in the number of inflammatory neutrophils (Fig. 2G) and eosinophils (not detected) were observed. Taken together, ME mice exhibited spontaneous respiratory dysfunction in mature adults (26-weeks old), with larger air spaces that progressed with age. In addition, the number of basophils, but not neutrophils, increased in the lungs.

### ECM proteins are highly related to the composition of emphysematous lungs in ME mice

**Figure 3:**
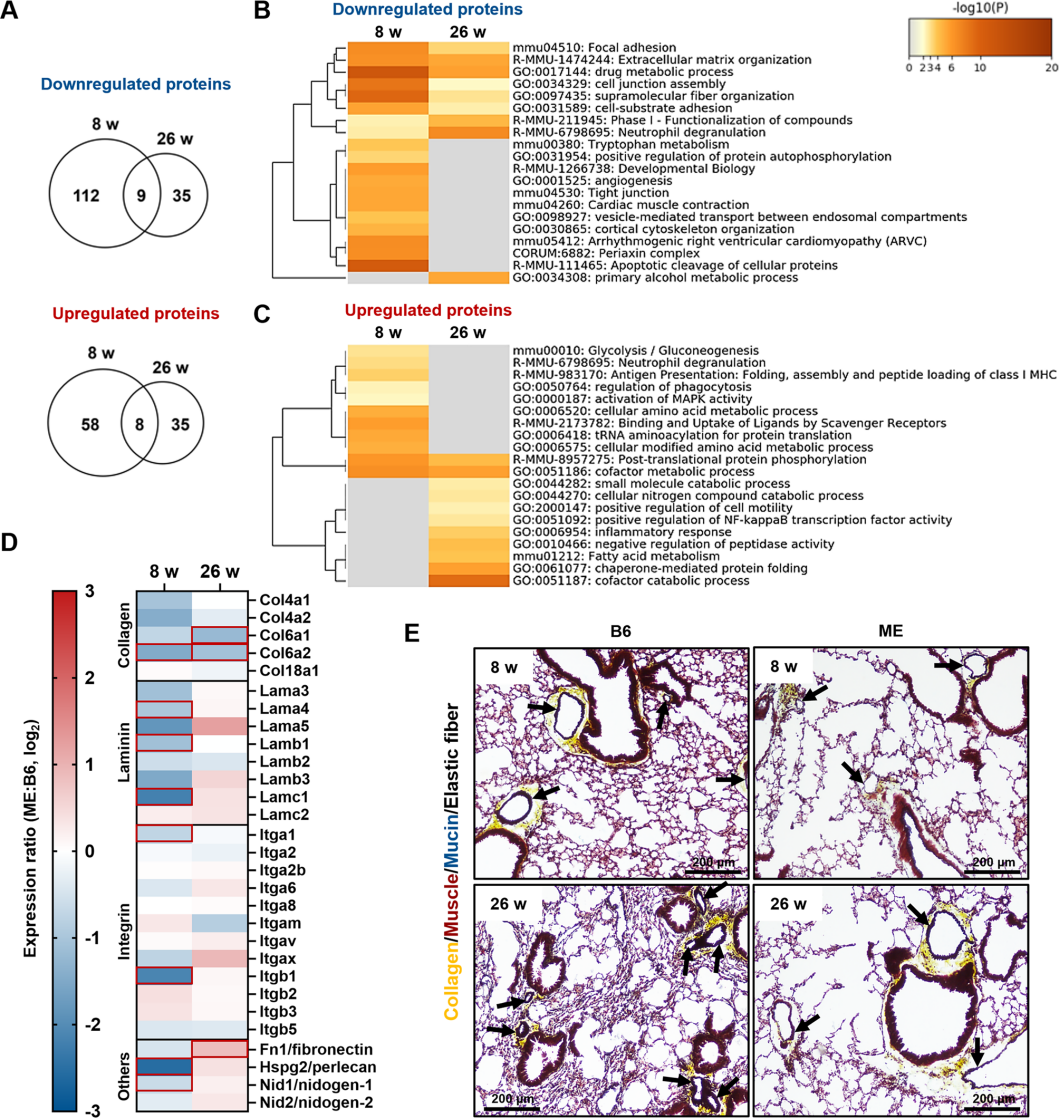
Proteomic analysis of protein expression in murine lung tissues. (**A**) Venn diagram of downregulated or upregulated proteins in ME mice between 8 and 26 weeks. (**B** and **C**) Gene ontology enrichment analysis revealed (**B**) downregulated and (**C**) upregulated protein clusters in ME mice. (**D**) Protein expression in the extracellular matrix component proteins detected in the lungs of B6 and ME mice. (**E**) Histology staining of lung extracellular matrix proteins using Movat pentachrome staining (collagen, yellow; elastic fibers, black; muscle, red; mucin, blue; fibrin, bright red). Arrows indicate locations of the artery with arterial collagen. Scale bar, 200 μm.

We also expected ME mice to have distinct lung proteome when compared to that in healthy B6 mice. Proteomic profiling of murine lungs revealed that a few hundred lung proteins were significantly different between B6 and ME mice at 8- and 26-weeks of age (Fig. 3A). However, only 9 downregulated and 8 upregulated proteins were consistent between the 8- and 26-week-old mice (Fig. 3A). Proteomic enrichment analysis was performed to determine the clusters of down- or upregulated proteins (Fig. 3B,C). Cluster analysis revealed that five ECM proteins (focal adhesion, extracellular matrix organization, cell junction assembly, supramolecular fiber organization, and cell-substrate adhesion) were consistently downregulated at 8- and 26-weeks of age (Fig. 3B). Individual values of the detected major lung ECM proteins (including not significant) in B6 and ME mouse lungs are shown in Fig. 3D. Although lung ECM proteins tended to decrease in ME mice when compared with that of B6 mice at 8-weeks of age (Fig. 3D), ME mice maintained equivalent amounts of ECM proteins similar to that of B6 mice at 26 weeks, except for a decrease in collagen (Fig. 3D). Movat pentachrome staining revealed that emphysematous lungs exhibited an artery decrease with collagen at 26 weeks of age (Fig. 3E). However, the large arteries retained their collagen (Fig. 3E). Furthermore, proteomic analysis showed a decrease in the cell signatures of alveolar type I cells and pericytes between B6 and ME mice lungs at 8- and 26-weeks of age (Supplementary Fig. S1A). The number of alveolar capillaries and thin walls consisting of alveolar cells decreased at 26-weeks of age due to emphysema (Supplementary Fig. S1B). In addition, we used transcriptome analysis to investigate different gene expression patterns in the lung cells of ME mice at 8 weeks, which is prior to emphysema development. We found 173 downregulated genes and 60 upregulated genes in ME mice when compared with those in the B6 mice (Supplementary Fig. S1C). Decreased gene and protein expression were consistent for eight terms, including the cytoskeletal and muscular terms; however, none of the upregulated terms were consistent (Supplementary Fig. S1D,E). Collectively, the downregulation of ECM protein clusters, such as arterial collagens, and a decrease in alveolar cell signature markers constituted molecular characteristics of the lungs of ME mice.

### Emphysematous murine lungs show significantly decreased EFEMP2 protein levels

**Figure 4:**
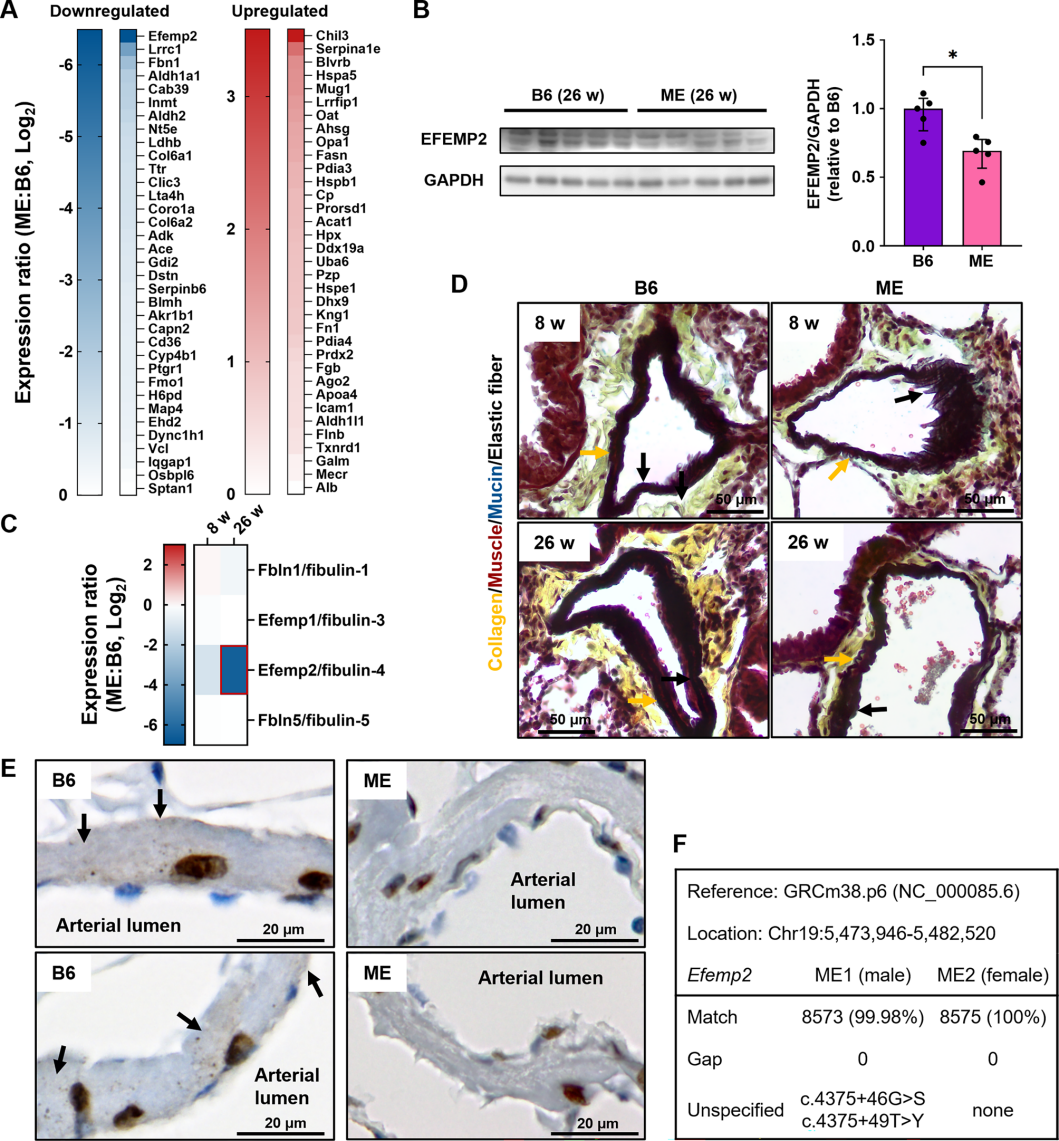
EFEMP2 protein expression and localization. (**A**) Identification of differentially expressed proteins between B6 and ME mice at 26 weeks. (**B**) Evaluation of EFEMP2 protein levels in lung tissues in B6 and ME mice at 26 weeks. Blots for EFEMP2 and GAPDH were exposed using different membranes that were transferred from the same gel. Data are calculated using the ImageJ software with 16-bit grayscale raw images and presented as the median with interquartile range (5 biological replicates, two-tailed Mann–Whitney test). *P < 0.05. (**C**) Protein expression in lung fibulin family proteins between B6 and ME mice. (**D**) Elastic fiber detection of lung arteries by Movat pentachrome staining (collagen, yellow; elastic fibers, black; muscle, red; mucin, blue; fibrin, bright red). Arrows indicate elastic fibers in the internal elastic lamina (black arrow) and external elastic lamina (yellow arrow). Scale bars, 50 μm. (**E**) Comparison of EFEMP2 levels in the lung artery. Arrows indicate locations of murine arterial EFEMP2. Scale bars, 20 μm. (**F**) Genome sequencing and comparison to the reference sequence of B6 for detection of *EFEMP2* gene variants of ME mice (male, n = 1; female, n = 1).

We then focused on each protein that was significantly different in the lung proteome between B6 and ME mice. Proteomic analysis revealed that Serpina1e, an ortholog of alpha-1-antitrypsin, deficiency of which leads to emphysema by disrupting elastin^26,27^, was ranked second among the significantly increased proteins when compared with the 26-week-old B6 mice (Fig. 4A). Another serine proteinase inhibitor protein, Serpinb6, which targets cathepsin G, decreased in ME mice (Fig. 4A). To identify the ECM component most affected during lung destruction, we searched for ECM proteins that were significantly downregulated. EFEMP2, which belongs to two ECM clusters (ECM organization and supramolecular fiber organization), was the most downregulated protein at 26-weeks of age (Fig. 4A). We confirmed the downregulation of EFEMP2 in the lungs of 26-week-old ME mice via immunoblotting (Fig. 4B). However, EFEMP2 was not downregulated at 8-weeks of age (Supplementary Fig. S2A,B), and the other detected lung fibulins were not downregulated at 8- and 26-weeks of age (Fig. 4C). Murine EFEMP2 is an essential ECM glycoprotein for collagen^28^ and elastic fiber^29,30^ synthesis. However, the retained collagen and elastic fibers of the arteries, which are formed during early postnatal development^31^, were not disrupted without any noticeable damage at 26 weeks of age (Fig. 4D). IHC staining was performed to determine the localization of intrapulmonary EFEMP2. We found that EFEMP2 was present in the nuclei and modestly detected in the pulmonary arteries of B6 mice, but much lesser in the arteries of ME mice (Fig. 4E). However, no genetic variants were found in the *EFEMP2* gene of ME mice (Fig. 4F). Pulmonary EFEMP2 levels decreased with age in ME mice.

### EFEMP2 protein is downregulated in emphysematous lungs of patients with COPD

**Figure 5:**
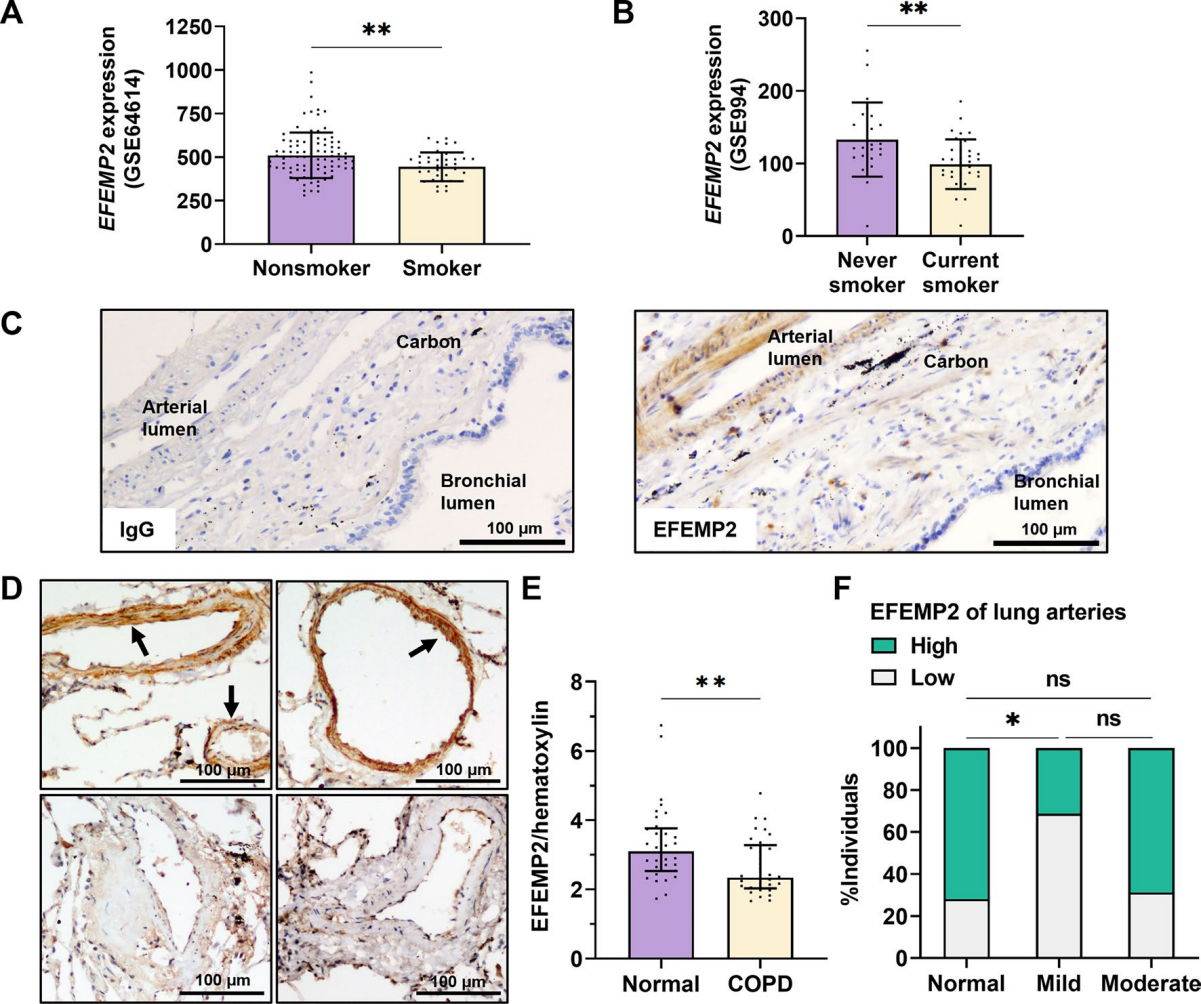
Gene expression and protein expression analysis of human EFEMP2. (**A**) *EFEMP2* gene expression in cells obtained from the small airway epithelium (GSE64614). Data are presented as the mean ± SD (nonsmoker, n = 91; smoker, n = 37, two-tailed unpaired t test). (**B**) *EFEMP2* gene expression in cells obtained from the bronchial epithelium (GSE994). Data are presented as the mean ± SD (never smoker, n = 23; current smoker, n = 34, two-tailed unpaired t test). (**C**) Representative human lung section image of IHC for EFEMP2. Control IgG using the same patient sample. A rabbit IgG antibody was used under the same w/v concentration and condition for EFEMP2. Scale bars, 100 μm. (**D**) Representative comparison of IHC images for high and low EFEMP2 expression in lung arteries and the alveolar area. Arrows indicate locations of human pulmonary EFEMP2 in the artery. Scale bars, 100 μm. (**E**) Measurement of EFEMP2 protein levels in lung section images based on ImageJ analysis. Data are presented as the median with interquartile range (n = 32 per group, two-tailed Mann–Whitney test). (**F**) Patient populations with high or low EFEMP2 levels based on IHC scoring of lung artery in the alveolar area (normal, n = 32; mild and moderate, n = 16, Chi-square/Fisher’s exact test with Benjamini–Hochberg false discovery rate procedure for multiple comparisons). ns, not significant. *P < 0.05, and **P < 0.05.

Structurally, murine and human EFEMP2 share 95% amino acid sequence similarity, with the only differences being amino acid substitutions and no insertions or deletions, thereby indicating similar biological functions^32^. Homozygous and compound heterozygous variants of the human *EFEMP2* gene result in aberrations in vascular elastogenesis^33^. However, the relationship between EFEMP2 expression and smoking status is unknown. We evaluated *EFEMP2* gene expression in human cell data that were classified based on smoking history using the small airway epithelium (GSE64614) and bronchial epithelium (GSE994) datasets in the GEO DataSets database. Smokers exhibited decreased *EFEMP2* expression in the small airway epithelium when compared to non-smokers (Fig. 5A). In addition, current smokers showed downregulated *EFEMP2* gene expression in the bronchial epithelium (Fig. 5B). Next, we evaluated EFEMP2 expression in human lungs surgically resected for tumor removal at the Shimane University Hospital. The data on the preoperative pulmonary evaluation concerning the individuals included in the study is presented in Supplementary Table S1. Pack-year of patients with mild COPD was higher than that of patients without COPD (Supplementary Fig. S3). In the human lungs, EFEMP2 was most frequently detected in the pulmonary arteries (Fig. 5C) and interpersonal differences in EFEMP2 levels were observed (Fig. 5D). To evaluate the individual differences in pulmonary EFEMP2 levels according to smoking status, we performed ImageJ analysis. The mean level of pulmonary EFEMP2 decreased in patients with COPD (Fig. 5E). Furthermore, we measured the amount of EFEMP2 in the arteries of the alveolar area by microscopic observation. Although patients with moderate COPD exhibited no differences when compared to those without COPD, we found significantly reduced EFEMP2 expression in the lung arteries of patients with mild COPD (Fig. 5F).

## Discussion

During airway remodeling in COPD, ECM components progressively decompose via inflammatory, oxidative, and/or proteolytic breakdown^10,34^. New findings regarding lung molecular information in animal models of emphysema, similar to those in humans, may be useful. This is the first study to examine lung molecules in ME mice that exhibited spontaneous emphysema that progressed and FEV0.05/FVC that declined with age from 8 to 26 weeks. Our results revealed lung ECM degradation of collagen and one ECM glycoprotein, EFEMP2, which was located in the pulmonary arteries and displayed a short-term (18 weeks) decrease in its levels. The low consistency of the proteome profiles of ME mice between 8- and 26-weeks of age suggests a radical molecular turnover in the lungs of ME mice during this period, thereby indicating a phenotype of accelerated aging. However, the mechanisms underlying low-inflammatory ECM deficiency during maturation remain to be elucidated. Based on previous reports of murine heterozygous *EFEMP2* variants resulting in low EFEMP2 expression and alveolar degradation^35^, we considered pulmonary EFEMP2 deficiency as one of the characteristics of the lungs in ME mice.

In humans, *EFEMP2* germline variants are known to cause autosomal recessive cutis laxa syndrome, presenting with cutis laxa, vascular tortuosity, and non-smoking early-onset emphysema^36^. Moreover, EFEMP2 level decreases in the aortic wall in patients with acute ascending aortic dissection, which is also associated with emphysema^37^. These results suggest that arterial EFEMP2 deficiency is a causative factor of emphysema. To the best of our knowledge, the present study is the first to show that *EFEMP2* expression in the pulmonary arteries was downregulated in patients with mild COPD who did not have cutis laxa syndrome, but were former smokers. Our analyses using GEO DataSets also showed that smokers had lower *EFEMP2* gene expression in the small airway and bronchial epithelium than non-smokers. Smoking affects gene transcription through epigenetic alterations, such as DNA methylation^38^. Thus, these results imply that cigarette smoke exposure is negatively associated with *EFEMP2* expression. Furthermore, the heart can be directly affected by age-related ECM protein remodeling^39,40^. Right ventricular hypertrophy (RVH) is strongly associated with emphysema/COPD and observed even in COPD without pulmonary hypertension^41^. Aberrant cardiac ECM proteins during RVH development have been observed^42,43^, and cardiac EFEMP2 has been detected^44^ in both humans and mice. Age-related EFEMP2 deficiency might play a role in the mechanisms underlying RVH development. Overall, our findings suggest that pulmonary EFEMP2 expression is associated with emphysema in both humans and mice. Further investigation of the decrease in EFEMP2 expression is required to elucidate its contribution to the development of emphysema with and without cigarette smoke-mediated inflammation.

Due to the controversies regarding the similarity in disease phenotypes between humans and animal models of COPD^17^, evaluation of the various aspects of disease characteristics is necessary when selecting animal models. ME mice, an animal model reflecting the limited characteristics of COPD, exhibit respiratory dysfunction with emphysema, lower body weight with shorter survival, and a slight increase in mean blood pressure, which are associated with COPD symptoms^5–7^ and aging^4^. Unlike cigarette smoke exposure, aging alone in healthy C57BL/6J mice does not induce lung cell senescence, which can contribute to lung function impairment^45^. Thus, the indirect consequences of mild, accelerated aging may induce systemic symptoms in ME mice at the mature adult (26-week of age) stage. However, we did not observe an increase in inflammatory neutrophils, bronchial wall thickening, and mucus accumulation, which are common symptoms of COPD airway inflammation. These findings indicate that pulmonary inflammation in ME mice is not as substantial as that in other animal models developed using exogenous stimuli, such as cigarette smoke and lipopolysaccharide^18,46^. In addition, emphysematous lungs of ME mice exhibited panlobular-like emphysema, similar to alpha-1-antitrypsin deficiency-induced emphysema rather than smoking-induced emphysema. Although a decrease in pulmonary Serpinb6 was observed in the proteomic analysis, its efficacy in emphysema development is unknown^47^ and was not evaluated in this study. Oxidative and/or proteolytic alterations, rather than developmental impairment, may be important in lung homeostasis of ME mice because the lung structure of ME mice was once constructed with lung function comparable to that of healthy B6 mice at 8 weeks of age. Moreover, oxidative stress is involved in accelerated lung aging^48^. Therefore, oxidative alterations may be one of the pathophysiological mechanisms in ME mice. Although we did not determine the critical inherited alterations involved in the pathogenesis, we plan to analyze genomic variants related to oxidative alterations and accelerated aging in ME mice.

Nevertheless, ME mice survived for approximately 70 weeks while walking a tightrope between healthy and lethal airway destruction. The survival period of ME mice with decreased respiratory function from 26-weeks of age onwards was > 50 weeks. Taken together, ME mice can be used in long-term studies on low-inflammatory alveolar destruction (> 6 months), such as finding preventive and curative treatments that begin before the onset of respiratory dysfunction, without expensive procedures to induce emphysema.

This study has certain limitations. The sample size was not large enough to clearly assess the disease phenotype of ME mice owing to individual differences in aging-related pathogenesis. Although we focused on non-tumor areas in lung specimens, we evaluated only the small outer areas of the specimens (two areas each) from patients with lung cancer, which could be different from those of patients without lung cancer who do not need to undergo surgical lung resection. In addition, we could only evaluate EFEMP2 in the remaining relatively large arteries, based on the decreased small alveolar areas. This may have been affected more in advanced emphysematous lungs.

## Conclusion

ME mouse is a model of mild, accelerated aging that exhibits low-inflammatory emphysema and respiratory dysfunction that develops at 26-weeks of age. Pulmonary EFEMP2 deficiency constitutes one of the molecular characteristics of emphysema in ME mice, similar to that observed in patients (those with mild COPD had less pulmonary arterial EFEMP2 than those without COPD).

## Methods

### Sample collection for mice

B6 (C57BL/6JJcl, CLEA Japan, Tokyo, Japan) mice and its inbred strain, ME mice were housed under conventional conditions with ad libitum access to food and water. Male B6 and ME mice without disabilities were not blindly allocated to the control and ME groups, respectively. Mice were anesthetized via intraperitoneal injection with a mixed anesthetic agent consisting of medetomidine (0.3 mg/kg), midazolam (4 mg/kg), and butorphanol (5 mg/kg). Blood was collected from the inferior vena cava. The blood was left in a 1.5 mL tube for 30 min and then centrifuged at 1500×*g* for 10 min at 4 °C. The supernatant was used as serum. After collecting the blood, an intravenous catheter was placed in the respiratory tract and held by tying strings. Bronchoalveolar lavage was performed by washing with PBS (500 μL for 8-week-old mice, 1 mL for 26-week-old mice) through the catheter. After washing thrice, the intrapulmonary cells in PBS were collected as the BALF. Then, the lungs were harvested and cut into several pieces for formalin fixation or frozen. A piece of the lung was fixed using 4% paraformaldehyde in PBS, and other pieces were frozen by immersion in liquid nitrogen and preserved at −80 °C for subsequent experiments. Sample groups of B6 and ME mice were not blinded through the experiments. Mice aged 4, 8, 26, and 56 weeks were considered as postnatal, young adult, mature adult, and middle aged, respectively^49^. All experiments involving animals were approved (No. IZ30-14) by the Animal Care and Use Committee of Shimane University, Izumo, Japan, and performed following the Guidelines for Proper Conduct of Animal Experiments by Science Council of Japan. This study was conducted in accordance with the ARRIVE guidelines and the Guidelines for Proper Conduct of Animal Experiments by Science Council of Japan.

### Blood pressure measurement

A non-preheating, non-invasive blood pressure monitor, Model MK-2000 (Muromachi Kikai, Tokyo, Japan), was used to measure tail-cuff blood pressure. Mice were caged in a Mouse Holder (Muromachi Kikai), and the tail was held by a tail-cuff (Muromachi Kikai). Systolic blood pressure, mean blood pressure, and calculated diastolic blood pressure were measured thrice, and the average value was used.

### Histological staining and quantification

Formalin-fixed murine lungs were embedded with paraffin as formalin-fixed paraffin-embedded (FFPE) specimens. FFPE sections were stained using hematoxylin and eosin staining solutions or the Movat Pentachrome Staining Kit (ScyTek, Logan, UT, USA). Image analysis was performed using ImageJ 1.53f. (National Institutes of Health, Bethesda, MD, USA). The binary image was generated from hematoxylin and eosin-stained lung tissue image using auto threshold of ImageJ, and then the MLI was calculated using Measure MLI plugin^50^. Distance transform watershed image from the binary image was obtained by Chamfer distance-based watershed using MorphoLibJ^51^ and Graylevel Watershed (http://bigwww.epfl.ch/sage/soft/watershed/) plugins. The area of each segment in the watershed image was used to calculate the equivalent airspace diameter D_2_^52,53^. The outer circle area (S) and the inner circle area (s) for the bronchial wall were measured using hematoxylin and eosin-stained lung tissue image. Bronchial wall thickness (r) was calculated using the following formula: r = √([S–s]/π).

### Pulmonary function measurement

Mice were anesthetized via intraperitoneal injection with a mixed anesthetic agent consisting of pentobarbital sodium (70 mg/kg) and xylazine hydrochloride (12 mg/kg). A cannula was placed into the respiratory tract and held by a sterilized tying string. The mice were kept in a flexiVent system (SCIREQ, Montreal, QC, Canada), and pulmonary function values were measured at a ventilator rate of 200/min and tidal volume of 10 mL/kg with a positive end-expiratory pressure of 3 cmH_2_O and a pressure limit of 30 cmH_2_O.

### Quantification of BALF

The BALF was centrifuged at 500×*g* for 5 min. The supernatant was discarded, and pellets were resuspended in a volume of PBS equal to that of the discarded supernatant. The suspension (400 μL) was cytocentrifuged with an EZ Single Cytofunnel with White Filter Cards (Thermo Fisher Scientific, Waltham, MA, USA) at 250×*g* for 5 min in a Cytospin 4 (Thermo Fisher Scientific). The white blood cells were deposited onto a cell deposition area that was 6 mm in diameter (28 mm^2^) on a glass slide and stained with Giemsa Stain Solution. The cell number per field of view (72 μm^2^) was counted using a BX53 (Olympus, Tokyo, Japan) with a 4 × lens and DP23 (Olympus). The total cell number was calculated using the following formula: Total cell number = cell number × (total suspension volume/400 μL) × (28 mm^2^/72 μm^2^). We observed 250 white blood cells on a slide to calculate the BALF cell population.

### Proteome mapping analysis for murine lung

Isobaric tags for relative and absolute quantification (iTRAQ) analysis were carried out to detect the difference in protein expressions. The collected lung tissues (30 mg per mouse) from six B6 or ME mice were mixed with a lysis buffer consisting of 50 mM Tris (pH 7), 0.5 mM EDTA, 20% glycerol, and 1% Protease Inhibitor Cocktail (Thermo Fisher Scientific). Each sample was sonicated in a 1.5-mL tube on ice with two cycles at 30% output for 10 s/cycle with a 5-s delay between bursts using the Branson Sonifier 450 (Emerson Electric, St. Louis, MO, USA). Samples were centrifuged at 13,000×*g* for 10 min at 4 °C, and the supernatants were collected as the protein lysates. The proteins were precipitated by adding five volumes of acetone at −30 °C and resuspended in 0.5 M triethylammonium bicarbonate (pH 8.5). The proteins were reduced, alkylated, trypsin digested, and labeled by iTRAQ reagents according to the iTRAQ protocol (Sciex, Framingham, MA, USA). The samples were desalted with a Strata-X 33 μm Polymeric Reversed Phase (Phenomenex, Torrance, CA, USA). Dried proteins (3 μg per sample) were analyzed by electrospray ionization mass spectrometry using a Prominence nano HPLC (Shimadzu, Kyoto, Japan) and the TripleTOF 5600 system (Sciex). Peptides were loaded onto a 3.5-μm Zorbax 300SB-C18 (Agilent Technologies, Santa Clara, CA, USA) and separated with a linear gradient of water/acetonitrile/0.1% formic acid (v/v). Spectral data were searched and analyzed using ProteinPilot 5.0 Software (Sciex) against *Mus musculus* (Mouse) in the SWISS-PROT Protein knowledgebase April 2017 (16,865 sequences).

### Microarray-based gene expression analysis

Murine lung tissue at 8 weeks (25 mg/tube), a 5-mm stainless ball, and 700 μL QIAzol Lysis Reagent (Qiagen, Hilden, Germany) were added to 2-mL tubes. The sample was homogenized twice using TissueLyser II (Qiagen) at 20 Hz for 5 min. The homogenized sample was used as the tissue lysate. Total RNA was extracted from the tissue lysate using the miRNeasy Mini Kit (Qiagen). Total RNA (10 μg per mouse) from six B6 or ME mice was mixed and used for the microarray analysis. Total RNA was labeled with Cy3 for B6 or Cy5 for ME by using the Amino Allyl MessageAMP II aRNA Amplification Kit (Thermo Fisher Scientific). Hybridization and microarray analysis for the expression of 23,474 genes was performed using 3D-Gene Mouse Oligo chip 24 k (Toray Industries Inc., Tokyo, Japan) and 3D-Gene Scanner (Toray Industries Inc.). Detected signals for each gene were normalized using global normalization method (Cy5/Cy3 ratio median = 1). Genes with a fold change of > 2 and < 0.5 were considered to be upregulated and downregulated genes, respectively.

### Transcriptome and proteome meta-analyses

Gene ontology enrichment of RNA or protein terms in upregulated or downregulated expression clusters was performed using Metascape (Database version: 2020-03-19)^54^.

### Immunoblotting

Murine lung tissue was mixed with 20 μL/mg T-PER Tissue Protein Extraction Reagent (Thermo Fisher Scientific) containing 1% Protease Inhibitor Cocktail (Thermo Fisher Scientific) and 1% Phosphatase Inhibitor Cocktail (Nacalai Tesque, Kyoto, Japan) in 1.5 mL tubes. The samples were sonicated on ice for three cycles at 30% output for 10 s/cycle with a 5-s delay between bursts using the Branson Sonifier 450 (Emerson Electric). The homogenized samples were centrifuged at 10,000×*g* for 5 min. The supernatants were collected as protein lysates, and protein concentration was quantified by Pierce Coomassie Plus (Bradford) Assay Kit (Thermo Fisher Scientific). Protein lysates were mixed with Bolt 4 × LDS Sample Buffer (Thermo Fisher Scientific) and Bolt 10 × Sample Reducing Agent (Thermo Fisher Scientific), and heated at 95 °C for 5 min. Each sample containing an equal total protein amount was electrophoresed on a 10% tris–glycine gel in a Bolt Mini Gel Tank (Thermo Fisher Scientific). Proteins on the gel were transferred to a 0.2-µm ClearTrans Nitrocellulose Membrane (Fujifilm Wako Pure Chemical, Osaka, Japan) using a Mini Blot Module (Thermo Fisher Scientific). The target protein was probed with primary antibodies against EFEMP2 (ab125073; lot: GR127962-8, 1:1,000, Abcam, Cambridge, England) and GAPDH (#8884; lot: 2, 1:2,000, Cell Signaling Technology, MA, USA). Anti-Rabbit IgG HRP-Linked Whole Ab Donkey (GE Healthcare UK, Buckinghamshire, England) was used as the secondary antibody. Blots were visualized using the ECL Select Western Blotting Detection Reagent (GE Healthcare UK) and LAS-4000 (Fujifilm, Tokyo, Japan).

### Immunohistochemistry and quantification analysis

The FFPE tissue slice (4 μm) was placed on a CREST glass slide (Matsunami, Kishiwada, Japan). Deparaffinization and antigen retrieval were performed using Ventana Benchmark XT (Roche, Basel, Switzerland) with Cell Conditioning Solution 1 (Roche). Anti-EFEMP2 antibody (ab125073; lot: GR127962-8, 1:200, Abcam) or anti-rabbit IgG antibody (R&D Systems, AB-105-C) diluted in Ventana Antibody Diluent (Roche) were added onto the slides manually and incubated at 37 °C for 1 h. The following secondary antibodies were used: SignalStain Boost IHC Detection Reagent (#8114, Cell Signaling Technology) for murine samples; ultraView Universal HRP Multimer (Roche) for human samples. 3,3′-Diaminobenzidine staining was performed with, DAB Chromogen, H_2_O_2_ (peroxidase activation, 37 °C for 8 m), and Copper (peroxidase inhibition, 37 °C for 4 m) of ultraView DAB Universal Kit (Roche). Counterstaining was performed with Ventana Hematoxylin II (Roche) and Bluing Reagent (Roche). Two non-overlapping areas comprising images of Immunohistochemistry (IHC) staining, including alveoli and arteries, but not tumor cells (300 × 300 μm/area), were imaged with a 10 × objective lens for each patient. The EFEMP2 level was measured in the IHC image using ImageJ and Colour Deconvolution plugin 3.0.3^55^ in the following order: subtract background (rolling ball radius = 100, light background, separate colors), color deconvolution (vectors = [H DAB]), invert, and then, mean of Colour_1 as EFEMP2, and mean of Colour_2 as hematoxylin were measured.

### Genomic DNA sequencing

Murine genomic DNA was extracted from the whole blood using the DNeasy Blood & Tissue Kit (Qiagen) and sequenced using a HiSeq X with 2 × 150-bp reads (Illumina, San Diego, CA, USA). Base calls and fastq generation were performed using Real-Time Analysis 2 (Illumina) and Bcl2fastq 2.20.0 (Illumina). Read filtering and mapping referenced with C57BL/6J genome assembly (GRCm38.p6) were performed using Trimmomatic 0.36^56^, BWA 0.7.17^57^, and Picard tools 1.111 (https://broadinstitute.github.io/picard).

### GEO datasets for human gene expression analysis

GEO datasets were downloaded from the Gene Expression Omnibus repository (https://www.ncbi.nlm.nih.gov/geo/).

### Patient specimens

Archived FFPE surgically resected human lung tissues of 64 former smoking patients, who had undergone surgical resection of lung adenocarcinoma in the right or left upper lobe of the lung between 2013 and 2019 at the Shimane University Hospital, were used. The patients had more than 70% forced expiratory volume in 1 s, and the forced vital capacity ratio in the preoperative pulmonary evaluation was included based on that of the normal group. The patients diagnosed with mild COPD or moderate COPD were included in the COPD group. The presence of emphysematous lesions in all patients of the COPD group was previously detected by pathologists using the FFPE samples during routine clinical examination. Informed consent in the period of follow-up care was obtained directly from patients. Informed consent of patients who ended the period of follow-up care was obtained in the form of an opt-out option through our website. The study protocol was approved (No. 4569) by the Research Ethics Committee of Shimane University, Izumo, Japan. The study was conducted in accordance with the principles of the Declaration of Helsinki.

### Pathological arterial protein analysis

A pathologist (N.M.), who had no information about the patients included in this study, examined two non-overlapping areas of the arteries in the alveolar region without tumor cells for each patient sample. The sample number and the sample groups of all slides were blindly randomized before the examination. The pathologist scored the EFEMP2 intensity of the artery on a scale of 0–3. The average value of each patient sample was used to categorize the sample as high intensity (> 1) and low intensity (≤ 1).

### Statistical analysis

For proteome mapping analysis, ProteinPilot 5.0 Software (Sciex) was used to calculate a *P*-value for each protein between B6 and ME mice at the same age. Other statistical analyses were performed using GraphPad Prism 9.4.0 (GraphPad Software, San Diego, CA, USA), except for the Benjamini–Hochberg false discovery rate procedure, which was performed using R Statistical Software (Foundation for Statistical Computing, Vienna, Austria). The experimental unit of the sample size for each group (n) represented a single animal or a human. A *P*-value < 0.05 was considered statistically significant. The tests used to compare groups and the post-hoc analysis are listed in the figure legends.

## Supplementary Information

Supplementary Information.

## Data Availability

Microarray data are available at the NCBI Gene Expression Omnibus (GSE188618).
