# Supplementary material for: Characterization of a spontaneous mouse model of mild, accelerated aging via ECM degradation in emphysematous lungs
Source: Sci Rep. 2023 Jul 3;13:10740. doi: 10.1038/s41598-023-37638-4 (PMC10317985; doi:10.1038/s41598-023-37638-4)

Characterization of a spontaneous mouse model of mild, accelerated aging via ECM degradation in emphysematous lungs

Ryosuke Tanino[1], Yukari Tsubata[1*], Takamasa Hotta[1], Tamio Okimoto[1], Yoshihiro Amano[1], Mayumi Takechi[2], Tetsuya Tanaka[3], Tomomi Akita[4], Mamiko Nagase[5], Chikamasa Yamashita[4], Koichiro Wada[6], and Takeshi Isobe[1]

[1]Department of Internal Medicine, Division of Respiratory Medicine and Medical Oncology, Faculty of Medicine, Shimane University, Izumo, Japan; [2]Department of Experimental Animals, Interdisciplinary Center for Science Research, Organization for Research and Academic Information, Shimane University, Izumo, Japan; [3]Department of Human Nutrition, Faculty of Contemporary Life Science, Chugoku Gakuen University, Okayama, Japan; [4]Department of Pharmaceutics and Drug Delivery, Faculty of Pharmaceutical Sciences, Tokyo University of Science, Noda, Japan; [5]Department of Organ Pathology, Faculty of Medicine, Shimane University, Izumo, Japan; [6]Department of Pharmacology, Faculty of Medicine, Shimane University, Izumo, Japan

***Correspondence**: Yukari Tsubata, Department of Internal Medicine, Division of Respiratory Medicine and Medical Oncology, Faculty of Medicine, Shimane University, 89-1 Enya, Izumo, Shimane 693-8501, Japan; Tel: +81-853-20-2580; Fax: +81-853-20-2581; E-mail: ytsubata@med.shimane-u.ac.jp.

Supplemental data

Supplementary figures S1, S2, and S3

Supplementary table S1

Immunoblotting pictures

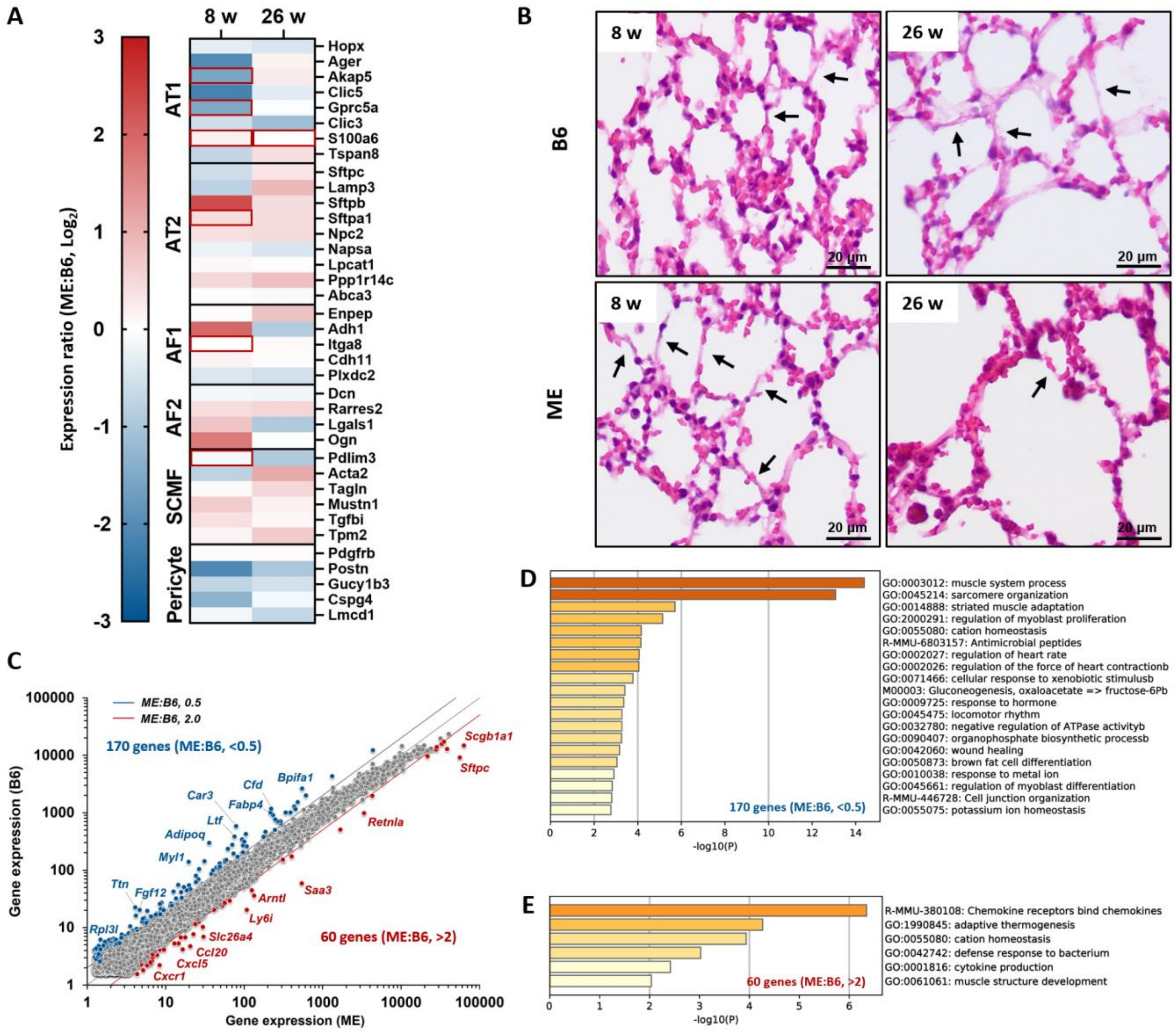

Supplementary figure S1. Proteomic, transcriptomic analyses and histological alveolar changes in the lung. (**A**) Protein expression of signature proteins for murine lung alveolar cells between C57BL/6J (B6) and Mayumi-Emphysema (ME) mice. AT, alveolar type; AF, alveolar fibroblast; SCMF, Secondary crest myofibroblast. The positive signature list of alveolar cells was obtained from LGEA Web Portal website (https://research.cchmc.org/pbge/lunggens/mainportal.html). (**B**) Histological changes in the alveolar septa. Arrows indicate the location of thin walls consisting of alveolar cells. Scale bars, 20 μm. (**C**) Identification of differentially expressed genes between B6 and Mayumi-Emphysema (ME) mice. Gene symbols of the top 10 genes among downregulated genes (ME:B6 < 0.5; blue) and upregulated genes (ME:B6 > 2; red) are shown. (**D** and **E**) Gene ontology enrichment analysis revealed (**D**) downregulated and (**E**) upregulated gene clusters.

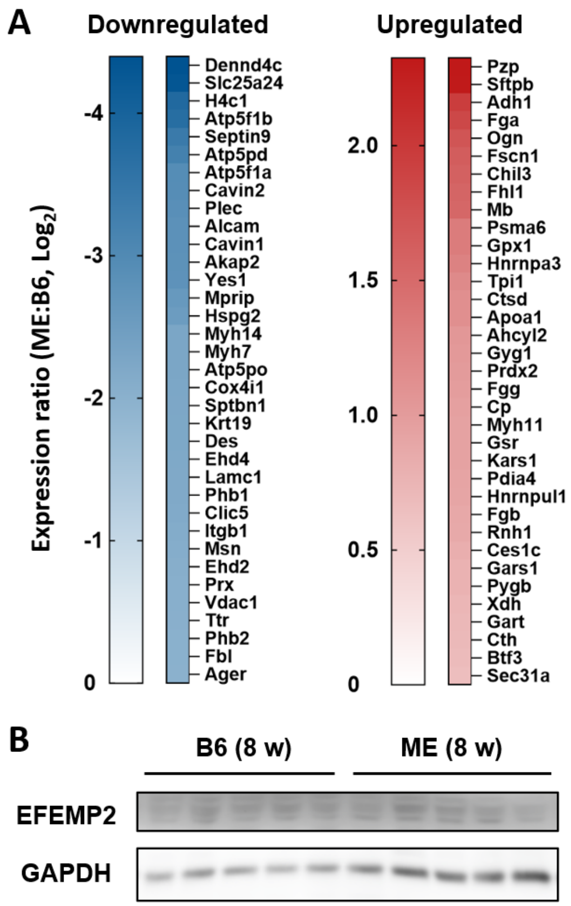

Supplementary figure S2. Protein expression in the lungs of young mice. (**A**) Identification of differentially expressed proteins between B6 and ME mice at 8 weeks. (**B**) Evaluation of EFEMP2 protein levels in lung tissues in B6 and ME mice at 8 weeks.

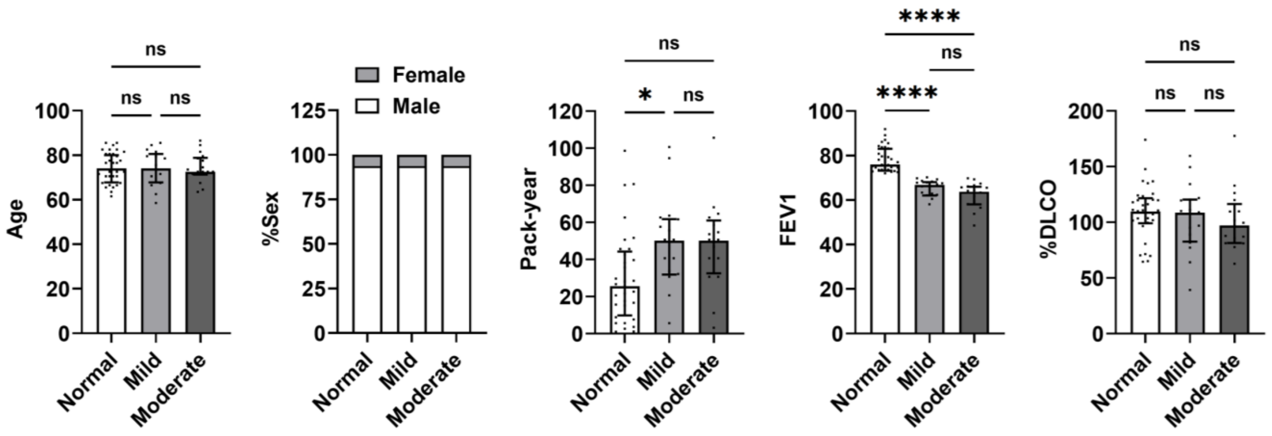

Supplementary figure S3. Comparison analyses of subject information among patient groups. Data are presented as the median with interquartile range (Normal, n = 32; Mild, n = 16; Moderate, n = 16, one-way ANOVA and Tukey's test for multiple comparisons). *P < 0.05, ****P < 0.0001, and ns, not significant. FEV1, forced expiratory volume in 1 s; DLCO, diffusing capacity of the lungs for carbon monoxide.

Supplementary table S1

Subject information of surgically resected lung for immunohistochemistry

Subject	Group	Age range	Sex	FEV1	Pack-year	%DLCO
1	Normal	80–89	Male	72.0	35.0	118.7
2	Normal	70–79	Male	71.5	21.0	102.4
3	Normal	80–89	Male	72.2	20.0	115.2
4	Normal	80–89	Male	72.4	2.0	135.8
5	Normal	70–79	Male	72.5	22.0	134.0
6	Normal	70–79	Male	73.0	42.0	173.1
7	Normal	60–69	Male	73.1	7.0	123.1
8	Normal	70–79	Male	73.1	17.0	114.3
9	Normal	80–89	Male	73.6	37.5	69.2
10	Normal	80–89	Male	74.1	62.0	98.6
11	Normal	70–79	Male	74.1	0.5	100.9
12	Normal	70–79	Male	74.3	8.3	146.7
13	Normal	70–79	Male	74.7	51.0	106.2
14	Normal	60–69	Male	75.0	15.0	109.5
15	Normal	70–79	Male	75.3	50.0	102.5
16	Normal	60–69	Male	75.4	28.0	110.1
17	Normal	60–69	Male	76.5	39.0	116.9
18	Normal	70–79	Male	77.6	98.0	109.7
19	Normal	80–89	Male	77.9	29.0	70.4
20	Normal	80–89	Male	78.8	26.0	107.5
21	Normal	70–79	Male	79.1	3.0	64.1
22	Normal	80–89	Male	79.1	45.0	63.6
23	Normal	80–89	Male	80.3	5.0	122.4
24	Normal	60–69	Male	82.7	5.0	124.6
25	Normal	60–69	Male	83.2	16.0	119.3
26	Normal	70–79	Male	83.3	14.5	108.9
27	Normal	70–79	Male	83.8	79.5	118.7
28	Normal	70–79	Male	85.6	25.0	79.5
29	Normal	70–79	Female	86.5	0.4	100.6
30	Normal	80–89	Male	88.6	27.5	136.1
31	Normal	60–69	Male	91.4	45.0	95.6
32	Normal	60–69	Female	85.0	80.0	68.7
33	Mild COPD	70–79	Male	69.3	94.0	87.9
34	Mild COPD	70–79	Male	69.7	50.0	109.1
35	Mild COPD	60–69	Male	62.4	30.0	107.9
36	Mild COPD	70–79	Male	61.9	55.0	76.2
37	Mild COPD	60–69	Male	67.4	31.5	133.3

(Table continues)

(Continued)

38	Mild COPD	80–89	Male	57.6	62.0	94.1
39	Mild COPD	70–79	Male	62.9	50.0	38.3
40	Mild COPD	50–59	Male	67.3	57.0	118.5
41	Mild COPD	70–79	Male	66.5	40.0	95.9
42	Mild COPD	70–79	Male	68.8	100.0	63.1
43	Mild COPD	60–69	Female	60.0	40.0	80.8
44	Mild COPD	80–89	Male	67.0	20.0	148.6
45	Mild COPD	80–89	Male	67.8	61.0	119.3
46	Mild COPD	70–79	Male	61.6	33.0	110.1
47	Mild COPD	70–79	Male	68.1	5.0	158.6
48	Mild COPD	80–89	Male	65.6	63.0	120.8
49	Moderate COPD	70–79	Female	63.8	30.0	80.0
50	Moderate COPD	60–69	Male	61.2	67.5	61.7
51	Moderate COPD	70–79	Male	62.9	53.0	100.6
52	Moderate COPD	70–79	Male	66.1	50.0	76.8
53	Moderate COPD	60–69	Male	56.0	10.5	108.5
54	Moderate COPD	70–79	Male	65.2	64.0	86.1
55	Moderate COPD	70–79	Male	57.7	46.3	80.2
56	Moderate COPD	70–79	Male	68.8	30.0	108.7
57	Moderate COPD	80–89	Male	58.9	62.0	118.9
58	Moderate COPD	80–89	Male	63.6	54.0	84.8
59	Moderate COPD	70–79	Male	53.3	50.0	86.8
60	Moderate COPD	70–79	Male	48.0	58.0	176.5
61	Moderate COPD	70–79	Male	66.9	40.0	95.5
62	Moderate COPD	70–79	Male	65.6	40.0	125.2
63	Moderate COPD	60–69	Male	69.3	105.0	98.5
64	Moderate COPD	80–89	Male	65.0	2.5	131.9

FEV1, forced expiratory volume in 1 s; DLCO, diffusing capacity of the lungs for carbon monoxide

Immunoblotting pictures

Immunoblotting picture for Figure 4B

Lysate samples were loaded onto a Tris-glycine gel for transfer to Membranes 1 and 2. Membrane placement was determined based on the position of visible markers loaded on the gel. One replicate was added to each end of both the groups.

Membrane 1 (>45kDa) with anti-EFEMP2 (55 kDa)

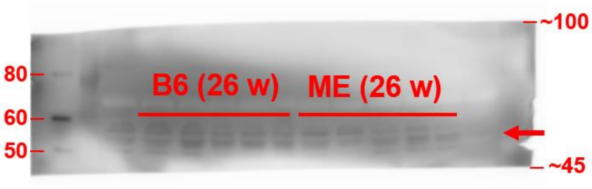

Membrane 2 (<45kDa) with anti-GAPDH (37 kDa)

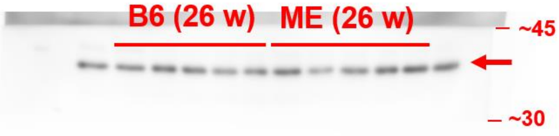

Immunoblotting picture for Supplementary figure S2B

Lysate samples were loaded onto a Tris-glycine gel for transfer to Membranes 3 and 4. Membrane placement was determined based on the position of visible markers loaded on the gel. One replicate was added to each end of both the groups.

Membrane 3 (>45kDa) with anti-EFEMP2 (55 kDa)

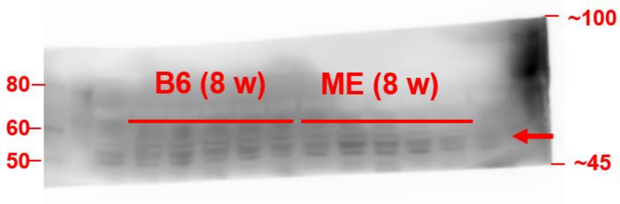

Membrane 4 (<45kDa) with anti-GAPDH (37 kDa)

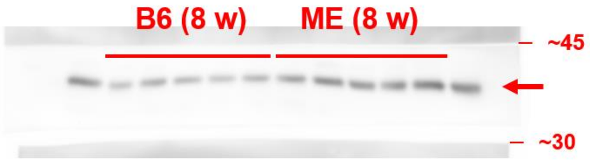

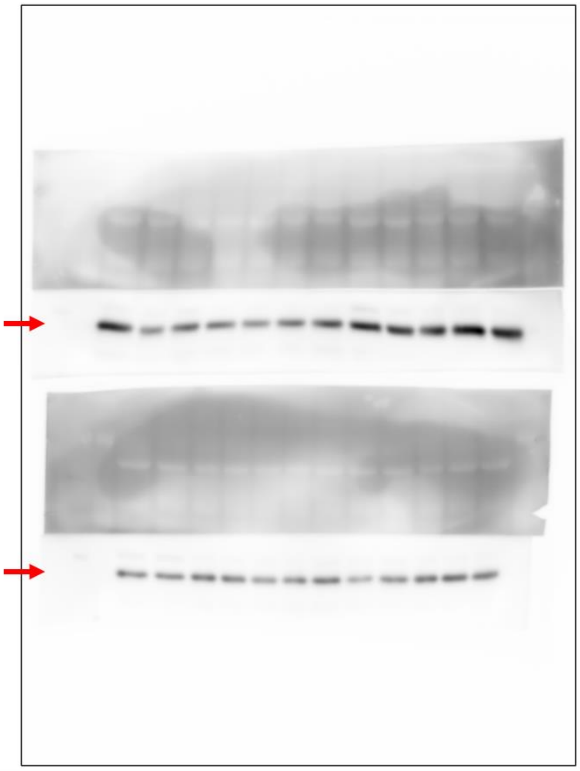

Full image and longer exposure images for GAPDH membranes

These membranes were imaged before the secondary antibody treatment because the HRP-conjugated anti-GAPDH antibody was used for Membrane 2 and 4.

Membrane 4 (longer exposure)

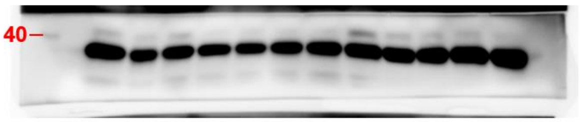

Membrane 2 (longer exposure)

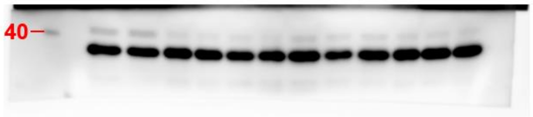

Supplement: Supplementary file 1 — Supplementary Information. [file 41598_2023_37638_MOESM1_ESM.pdf]
